# Potential for cervical cancer incidence and death resulting from Japan’s current policy of prolonged suspension of its governmental recommendation of the HPV vaccine

**DOI:** 10.1038/s41598-020-73106-z

**Published:** 2020-09-29

**Authors:** Asami Yagi, Yutaka Ueda, Satoshi Nakagawa, Sayaka Ikeda, Yusuke Tanaka, Masayuki Sekine, Etsuko Miyagi, Takayuki Enomoto, Tadashi Kimura

**Affiliations:** 1 Department of Obstetrics and Gynecology, Osaka University Graduate School of Medicine, 2-2, Yamadaoka, Suita, Osaka 565-0871 Japan; 2 Department of Gynecology, International University of Health and Welfare Mita Hospital, 1-4-3 Mita, Minato-ku, Tokyo, 108-8329 Japan; 3 Department of Obstetrics and Gynecology, Niigata University Graduate School of Medical and Dental Sciences, 1-757 Asahimachi-dori, Chuo-ku, Niigata, 951-8510 Japan; 4 Department of Obstetrics and Gynecology, Yokohama City University Graduate School of Medicine, 3-9 Fukuura, Kanazawa-ku, Yokohama, Kanagawa 236-0004 Japan

**Keywords:** Environmental social sciences, Health care, Oncology, Risk factors

## Abstract

In 2013, recurrent reports of diverse symptoms occurring in girls after receiving HPV vaccination appeared in Japanese media. The Ministry of Health, Labor and Welfare quickly responded by announcing a temporary suspension of its recommendation for the vaccine. The HPV vaccination rate soon fell to almost zero. In the present study, we calculated the potential future numbers of cervical cancer incidence and death that will be increased by this policy decision. We have assumed that the number of yearly vaccinations is evenly distributed across a daily basis. Future incidence and death increased in females born in FY2000 are estimated to be 3651 and 904, respectively, 4566 and 1130 for those born in FY2001, 4645 and 1150 for those born in FY2002, and 4657 and 1153 for those born in FY2003. In FY2020, the large increase of risks to females born in FY2004 amounts to 12.0 females per day who will now be at a higher risk for acquiring of cervical cancer in their future, and 3.0 females per day newly at risk for future death from that disease in its progressive form. No one should be able to accept this situation. We sincerely ask the government to resume its recommendation for the vaccine as soon as possible.

## Introduction

In May of 2018, the Director-General of the World Health Organization (WHO) announced a global call-to-action towards the elimination of cervical cancer^1^. In January of 2019, a global strategy for doing just that was announced at the 144th Session of the WHO Executive Board^2^. They listed the known barriers to the worldwide elimination of cervical cancer, such as a Human Papilloma Virus (HPV) vaccine shortage, price (especially in middle-income countries), vaccine hesitancy, etc.

In Japan, a stalled policy decision, initiated in 2013 by the Ministry of Health, Labor and Welfare (MHLW) to suspend its governmental recommendation for HPV vaccination, has become a major health issue. A brief history of the current situation for HPV vaccination in Japan shows that public subsidies for the Human Papilloma Virus (HPV) vaccine started in Fiscal Year (FY) 2010, when females aged 13–16 could be immunized with only a small out-of-pocket fee. The HPV vaccination rate soared to nearly 70% in targeted females^3–5^. By April of 2013, HPV vaccination had become a ‘national routine immunization’, meaning that thereafter females aged 12–16 could receive the vaccine for free.

With the success of such massive numbers of immunizations, problems were sure to evolve. There began to appear repeated media reports of a diverse group of symptoms occurring in young girls after their HPV vaccination. In an understandable precautionary response, the MHLW announced a temporary suspension of its recommendation for the vaccine in June of the same year. The HPV vaccination rate in Japan soon fell to almost zero, where it has since languished^3–5^. This has led to immunization rates in Japanese females that stretch from near 70% to nearly 0%, depending completely on a girl’s FY of birth.

Females born in Japan in 1993 or earlier were not vaccinated for HPV, as the vaccine had not yet been introduced, and public subsidies did not begin until FY2010, by which time even the youngest of them were already 17. Females born from 1994 to 1999 were immunized under public subsidies for the vaccine, and achieved a 70% vaccination coverage rate for this cohort. Following the MHLW’s negative policy change in 2013, the vaccination rate reverted to nearly zero for girls born in 2000 and thereafter, as they refused to be vaccinated under the cloud of suspicion surrounding its adverse events.

We have previously calculated the relative risk of infection with HPV-16/18 at age 20, and the resulting lifetime incidence of HPV-caused cervical cancer, and we predicted these risks would differ greatly in Japan, depending on a girl’s FY of birth—because of the lapse of protective vaccinations that has occurred since June of 2013^6,7^. For example, because the recommendation of HPV vaccine was not restarted at the end of FY2019, those females born in 2000, 2001, 2002 and 2003 are now over the targeted ages of 12–16. It is not realistic to think that these unvaccinated girls will never experience sexual intercourse. We can say that the risk for these females is ‘fixed in place’. If the resumption of the recommendation is delayed for one more year, the relative risk of future HPV-16/18 infection in females born in FY2004 in Japan will be at least like the risks that existed before the HPV vaccine was first introduced.

Here, we have estimated the future daily numbers of cervical cancer incidence and death increased by the stalled policy decision of the suspension of the governmental recommendation for the HPV vaccine.

## Methods

The data used in the present study was modified from those shown in the MHLW council meeting^8^ to reflect the real cumulative vaccination rate for each birth FY^9^. In summary, the cumulative initial vaccination rate for each birth year: Born in FY1994: 55.5%, Born in FY1995: 73.5%, Born in FY1996: 78.2%, Born in FY1997: 78.8%, Born in FY1998: 78.7%, Born in FY1999: 68.9%, born in FY2000: 14.3%, born in FY2001: 1.6%, born in FY2002: 0.4%, born in FY2003: 0.2%, born in FY2004: 0.1%, born in FY2005: 0.0%. Notably, the vaccination rate for females born in FY2000 has been corrected from 42.9% to 14.3%.

The relative risk of cervical cancer incidence was calculated using conditions from our previous predictions^7^, modified as follows: (1) One vaccination shot is considered to be sufficiently effective for preventing HPV infection (this decision was needed because in most cases only data for the first inoculation could be obtained). (2) Lifetime incidence risk is proportional to lifetime HPV infection risk. (3) Cervical cancer is caused predominantly by HPV infection, and of them, the proportion caused by high-risk HPV-16/18 sub-type infections (targeted by the most common vaccine) is 60%. HPV-16/18 sub-types and other types of mixed infection are prevented by the vaccine. (4) Even when vaccination is given, the incidence of cervical cancer caused by HPV other than HPV-16/18 will not change. (5) The population per birth year does not change significantly in Japan. (6) Unless the vaccine is present, the incidence of cervical cancer does not change. (7) The presence or absence of vaccination and sexual activity are independent of each other, and sexual activity does not increase significantly following vaccination (unpublished findings). (8) The number of yearly vaccinations is evenly distributed across a daily basis. (9) A female who receives vaccination and experiences sexual intercourse in the same year is vaccinated before having that sexual intercourse. (10) HPV infection risk is calculated based on the rate of having sexual intercourse without HPV vaccination at every age, and over a lifetime. (11) Sexual activity and cervical cancer prevention behaviors of the females who are over the target age without vaccination don't change. (12) In the case of when a governmental recommendation is resumed, within the resumed year the vaccination rate for each birth year will recover to 68.93%, the same as the vaccination rate for those born in FY1999. (13) The rate of experiencing sexual intercourse at the age of 12, 13, 14, 15, 16 and 17 is assumed to be 0%, 1%, 2%, 5%, 15%, and 25%, respectively. The rate of experiencing sexual intercourse throughout a lifetime is assumed to be 85%^10^.

We have also assumed that the number of people suffering from cervical cancer and the number of deaths will remain unchanged by any conditions other than the vaccination rate. The increased numbers of future incidences of cervical cancer and death, in each birth FY, compared to those of females born in FY1999, most of whom received HPV vaccination with public subsidies before the suspension of the recommendation, were calculated by multiplying the increased relative risk, compared to the risk for females born in 1999, by the number of affected females, 11,293, in the latest statistics for FY2014, and the number of deaths, 2795, in the latest statistics for FY2017^11^.

All methods were analysis of statistical data not obtained from human participants (including the use of tissue samples), and informed consent was not required. The study was performed in accordance with the relevant guidelines and regulations. All the data used in the present analysis were publicly available or derived from previous publications shown above.

### Ethical statement

This study was approved by the Institutional Review Board and Ethics Committee of the Osaka University Medical Hospital.

## Results

**Figure 1:**
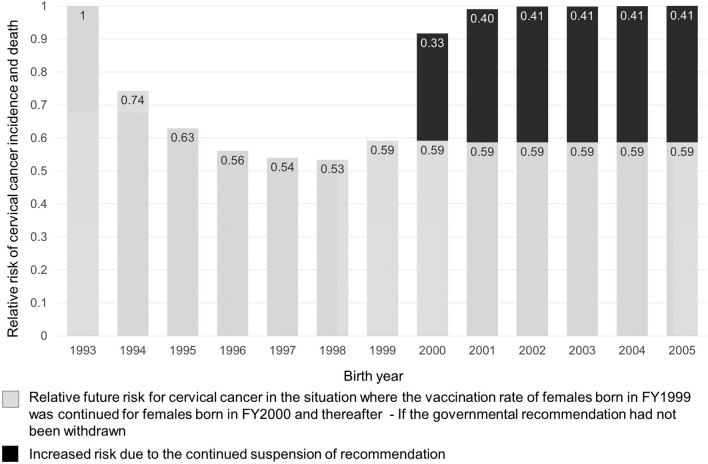
Estimated number of future cervical cancer mortalities and deaths generated daily. We set the future risk of cervical cancer incidence and death in females born in FY1993 as the baseline of 1 (these females were never vaccinated, because they were over-aged (> 16) for receiving HPV vaccination when public subsidies started in FY2010). FY: fiscal year.

Theoretically, if the suspension of the governmental recommendation hadn't been implemented, and if the vaccination rate of females born in FY1999 had continued, at the same rate as then, with all females born thereafter, then the relative risk of cervical cancer incidence and death for each birth FY would have remained flat, at 0.59 (Fig. 1). However, in reality, due to the suspension of the recommendation that did occur, the relative risk rose dramatically because the vaccination rate was extremely low in females born in and after FY2000. For females born in FY2000, the estimated value of the increased relative risk for future incidence and death was an additional 0.33. It was 0.40 for those born in FY2001, and 0.41 for those born in and after FY2002.

**Figure 2:**
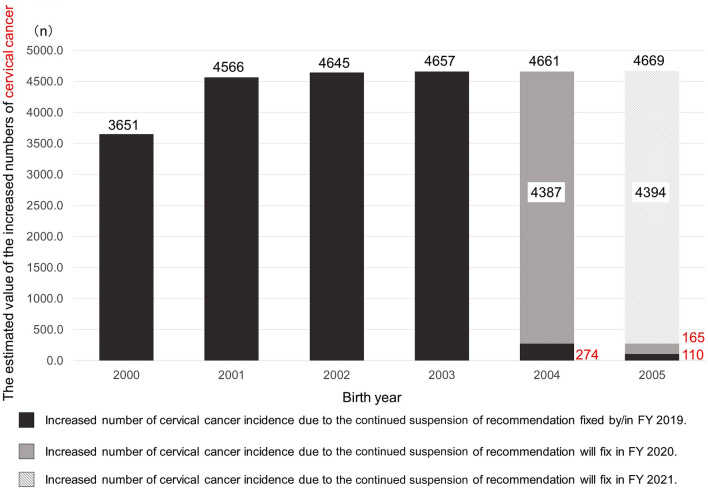
The estimated increased numbers of cervical cancer patients. As an example, the estimated increased numbers of cervical cancer patients fixed in FY2019 among those born in FY2003 was calculated as follows: 1. Relative risk of cervical cancer among females born in FY2003—if the governmental recommendation restarted in FY2019 = (Rate of cervical cancer that cannot be prevented with HPV vaccine) + (Rate of cervical cancer that can be prevented with HPV vaccine) × (Rate of girls unprotected by HPV vaccine who were born in FY2003)/(Rate of girls unprotected by HPV vaccine who were born in FY1993. 2. Rate of girls unprotected by HPV vaccine who were born in FY2003 = ( Non-inoculation rate at 13 years old) × (sexual experience rate at 13 years old) + (non-inoculation rate at 14 years old ) × (new sexual experience rate at 14 years old) + (non-inoculation rate at 15 years old) × (New sexual experience rate at 15 years old) + (Non-inoculation rate at 16 years old) × (New sexual experience rate at 16 years old) + (Non-inoculation rate during lifetime) × (New sexual experience rate during lifetime). 3. Rate of girls unprotected by HPV vaccine who were born in FY1993 = (Lifetime non-inoculation rate) × (Lifetime sexual experience rate). 1, 2, and 3. The rate = 0.4 + 0.6 × ((1–0.0012) × 0.01 + (1–0.0019) × (0.02–0.01) + (1–0.0019) × (0.05–0.02) + (1–0.6893) × (0.84–0.15)) / 1 × 0.85 = 0.6107. 4. Relative risk of cervical cancer to be fixed in place if the governmental recommendation is not resumed in FY2019 = (Relative risk of cervical cancer among those born in FY2003—if the governmental recommendation is resumed in FY2020, as calculated in the same way as 1, 2 and 3)—(Relative risk of cervical cancer among those born in FY2003—if the governmental recommendation is resumed in FY2019). The rate = 0.9998–0.6107 = 0.3881. 5. Number of females with cervical cancer fixed in place in FY2019 among females born in FY2003 = (Relative risk of cervical cancer to be fixed if the governmental recommendation is not resumed in FY2019) × (Nationwide cervical cancer cases estimated from the Regional cancer registry). The number = 0.3881 × 11,293 = 4383. Similarly, that fixed in place of FY2018 among those born in FY2003 was estimated to be 274, and the increased number for the females born in FY2003 was calculated to be 4657 in total.

**Figure 3:**
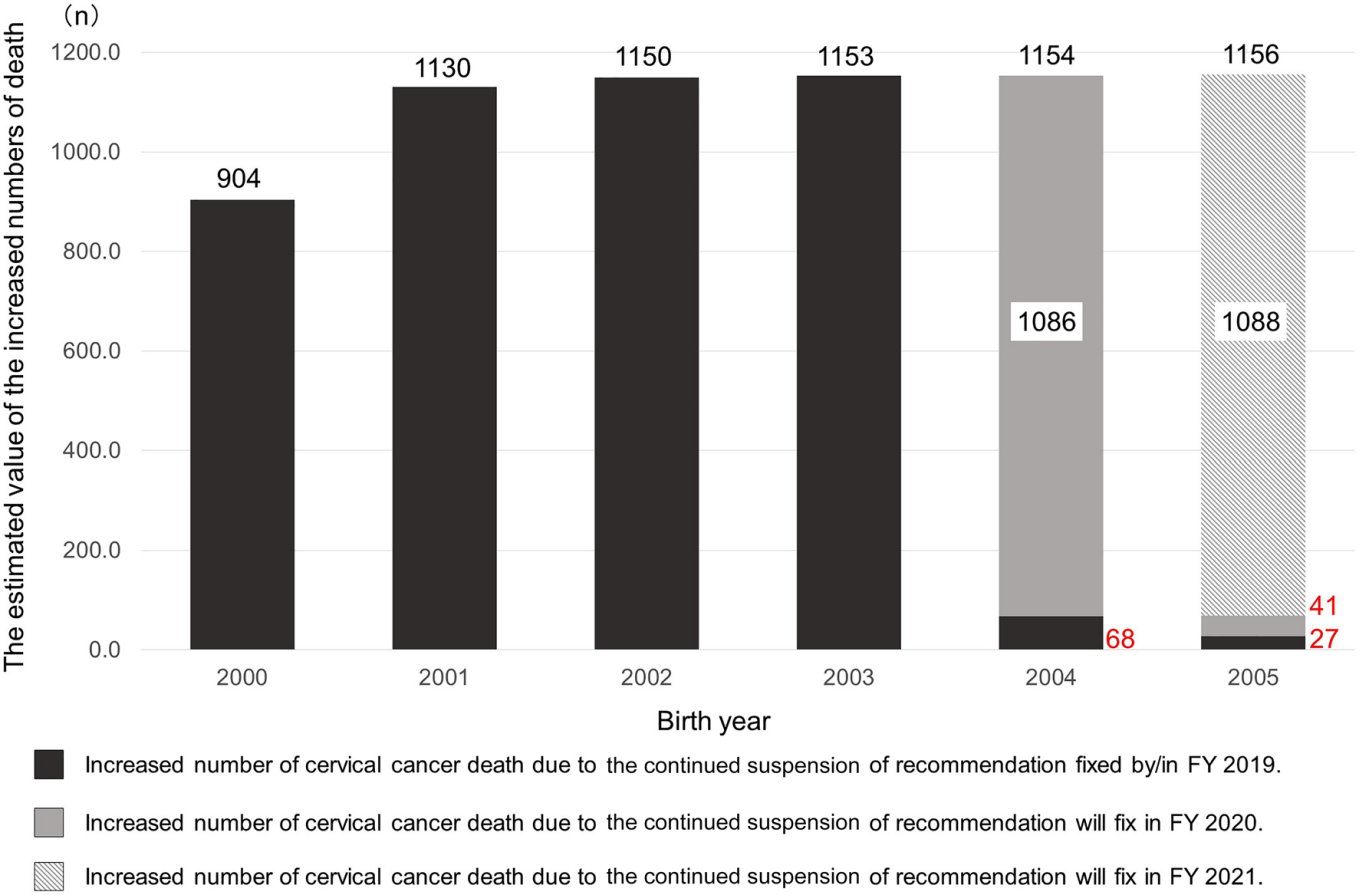
The estimated increased numbers of cervical cancer deaths.

The estimated value of the increased numbers of cervical cancer patients and deaths are depicted in Figs. 2 and 3, respectively. The estimated value of the increased numbers of cervical cancer patients and deaths among females born between FY2000 and FY2003 remained steady state because the governmental recommendation of HPV vaccination hadn’t resumed by FY2019. Moreover, in females born in and after FY2004, the increased number of cervical cancer patients and deaths is being / will be fixed in FY2020 and thereafter. In particular, in females born in and after FY2004, an estimated increase of 274 future cervical cancer cases was already fixed by FY2019, and an increase of 4,387 patients is being cemented in place during FY2020 (Fig. 2). In addition, as the resumption of the recommendation delays for another year, an increase of 4,669 patients will be fixed in females born in and after FY2005.

The same applies to the estimation of the increased numbers of cervical cancer deaths. The estimated value of the increased numbers of cervical cancer deaths among females born between FY2000 to FY2003 were fixed because the governmental recommendation of HPV vaccination didn't resume by FY2019 (Fig. 3). In females born in FY2004, an increase of 68 deaths was already fixed by FY2019, and an increase of 1,086 patients is being fixed in FY2020 (Fig. 2). As the resumption of the recommendation delays for one more year, an increase of 1,156 deaths will be fixed in females born in and after FY2005.

In FY2020, as the suspension of the governmental recommendation of HPV vaccination has dragged on, the large increase of risks to females born in FY2004 is being perpetuated on a daily basis because of a failure to get the protective HPV vaccination—as they will not suppress their sexual activity due to having refrained from HPV vaccination (Table 1). This amounts to 12.0 females per day who will now be at a higher risk for acquiring of cervical cancer in their future, and 3.0 females per day newly at risk for future death from that disease in its progressive form (Supplementary Information). Unless nationwide vaccination is resumed in FY2020, these estimated increases of future incidence and death from cervical cancer, 12.0 and 3.0 per day, will be fixed yet again, on a daily basis in FY2021, on those females born in FY2005.

**Table 1:** Increased future incidence and death for each birth FY.

		Will increase in 2020	
Birth year		2004	2005
Incidence	Per day	12.0	0.5
	Per month	365.5	13.7
	Per year	4386.5	164.8
Death	Per day	3.0	0.1
	Per month	90.5	3.4
	Per year	1085.6	40.8

The increased numbers of future incidence and death in each birth FY, compared to those of females born in FY1999 (most of whom received HPV vaccination with public subsidies before the suspension of the recommendation in FY2013) were calculated. The risk for females born in and after FY2006 could not be calculated because the vaccination rates were available only for those born in and before FY2005.

## Discussion

The WHO has expressed a deep concern about “the circumstances in Japan” and has declared that, “policy decisions based on weak evidence, leading to lack of use of safe and effective vaccines, can result in real harm”^12^. In spite of this condemnation from the world health body, Japan’s suspension of governmental recommendation for the HPV vaccine has continued. Tragically, this is resulting in ever larger apparent disparities in the vaccination rates of our children—depending on their birth year. Furthermore, the screening rate for cervical cancer in Japan is correspondingly extremely low^13^, which greatly compounds the risk for cervical disease progression to lethal states before detection.

Incredibly, in Japan, the cervical cancer screening rate for females in their 20′s is only 26%. The Japanese government's countermeasure for cervical cancer have clearly not been successful and Japan is lagging far behind countries of similar economic status. In stark contrast, the Australian National HPV Vaccination Program is going well. Their age-adjusted incidence rate of cervical cancer will be reduced to rare cancer levels by 2020^14^. One of the ways Australia has made this program so successful is their communication strategy, which included healthcare professionals, mothers, and the young girls targeted for vaccination, which began before the vaccine was even introduced. In particular, they worked on thorough risk communication.

In Ireland and Denmark, their HPV vaccination rates dropped dramatically after an adverse side-effects documentary broadcast by their media, as it had in Japan, but, unlike Japan, their vaccination rate has since recovered^15^. In Ireland, the Irish National Immunization Office established a steering group to actively promote the vaccine, and the HPV Vaccination Alliance was launched. The Alliance, consisting of a group of over 35 different organizations, developed a strong multi-media campaign, which included an effort called ‘Meet Laura Brennan’. Ms. Brennan was over the target age of 12–16 when the HPV vaccine was first rolled out in Irish schools. After receiving a diagnosis of stage IIB cervical cancer, she worked to publicly advocate in favor of the HPV vaccine. In Denmark, an information folder and a telephone hot-line were used to provide information and consultations, while keeping the emotions of parents in the forefront of their messaging, based on the concept that scientific data is usually trumped by emotions^16^. These two countries, which both worked quickly to respond to the side-effect health scare, have since fully recovered their pre-scare vaccination rates. It now seems that only Japan was left behind.

As for the initial reports about the diverse group of HPV vaccination symptoms, when the data from the large Nagoya and Sobue Studies were not yet available, it was reasonable and responsible journalism to report the potential health risks of the vaccine. However, we scientists and physicians now know, from those studies, that the HPV vaccine is as safe as any other vaccine we have and use. Under these circumstances, our medical professionals, media, politicians, MHLW, teachers, and parents are in a position to promote the health of those young women who have already missed their chance at HPV vaccination, and those that are about to.

In the present study, we have calculated the potential for increased numbers of future cervical cancer incidence and death due to the suspension of the governmental recommendation of the HPV vaccine, this helps us to realize a sense of urgency towards a looming crisis, a nation-wide loss of health and lives caused by the governmental decision to not restart its recommendation for a safe and effective vaccine. As each day passes, because of this decision, more and more eligible girls are not vaccinated, then they become age-ineligible, and are likely to remain unvaccinated as they begin their risk of sexual exposure to HPVs. Their risk of developing cervical cancer in the future will be needlessly high—unless their subsequent sexual activity and cancer screening behavior can be changed. This assumption is realistic, as young women are unlikely to suppress their sexual activity due to having refrained from HPV vaccination, and the national cervical cancer screening rate has been consistently low despite various efforts.

HPV vaccination dissemination among Japanese females born between 1994 and 1999 was achieving remarkable success—just before the announcement of the suspension of the governmental recommendation in 2013. By the first of FY2020, females born in 2000, 2001, 2002 and 2003 were already over 12–16, the targeted and subsidized ages for the vaccine. In FY2020, as the suspension of the governmental recommendation of HPV vaccination has dragged on, the large increase of risks to females born in FY2004 is being perpetuated on a daily basis.

One of the limitations of the present study is that potential for cervical cancer incidence and death was calculated using certain conditions, and we have to wait for the real data for long. We have previously predicted the relative risk of infection with HPV-16/18 at age 20, and the resulting lifetime incidence risk from cervical cancer, using the HPV vaccination rate in only one city^6,7^. In the present study, we have shown that risks of the females were already, is being, and will be fixed depending on the birth FY.

Recently, Simms K et al. reported the impact of HPV vaccine hesitancy on cervical cancer in Japan^17^. According to their modeling study, the vaccine crisis from 2013 to 2019 is predicted to result in an additional 24,600–27,300 cases and 5000–5700 deaths over the lifetime of cohorts born between 1994 and 2007, compared with if coverage had remained at around 70% since 2013. We focused on the risk of the birth FY in our present study. Because the cervical cancer risk would be increased in the females born in and after 2000, the additional number of cervical cancers and deaths over the lifetime in the females born from 2000 to 2007 could be calculated, from their report, to be around 3100–3400 (24,600–27,300/8) cases and around 600–700 (5000–5700 /8) deaths in each birth year. These numbers were similar to, but a little lower than the predicted in our study. One of the reasons is the difference in models used. They conducted a precise prediction by taking various factors into account using ‘Policy1-Cervix’, an extensively validated dynamic model of HPV transmission, vaccination, type-specific natural history, cancer survival, screening, diagnosis, and treatment. However, our estimation was a simple one. We just predicted the cumulative risk of cervical cancer using certain conditions that lifetime incidence risk is proportional to lifetime HPV infection risk and HPV infection risk is calculated based on the rate of having sexual intercourse without HPV vaccination.

Another possible reason is that we used the corrected vaccination rate in the females born in FY2000. The rate shown in the MHLW council was 42.9%, however, the rates was incorrectly calculated. We used the corrected rate, 14.2%, in our present study, and the risk of future cervical cancer incidence and death in those born in FY2000 was estimated to be higher than their estimation.

These unvaccinated females have now reached the age where sexual intercourse, and the risk of HPV exposure, naturally begins. Their future of acquiring and dying from HPV-caused cervical cancer is now a predetermined risk for them—unless they refrain from all sexual intercourse to avoid infection and cervical cancer – which, we can all agree on, is unrealistic. From this viewpoint, their future for incidence and death from HPV-related diseases is being predestined on a daily basis.

Who will take responsibility then for this tangible harm? We sincerely ask the government not only to resume its recommendation for the vaccine as soon as possible, but also to provide an easily accessible and highly affordable opportunity for immunization to those females who already missed getting immunized during the vaccine recommendation hiatus. Cervical cancer screenings should be also be more strongly recommended for these females, as they are at much higher risk. The nine-valent vaccine, which has not yet been introduced in Japan, and immunization for young boys, should both be strongly considered. With discontinuation of recommendations, it is important to keep in mind that the future risk of cervical cancer is rising every day in individual females. No one should be able to accept this situation.

## Supplementary information

Supplementary file1

## Abbreviations

FY: Fiscal year
HPV: Human papillomavirus
MHLW: Ministry of Health, Labor and Welfare
WHO: World Health organization
