# Supplementary material for: Potential for cervical cancer incidence and death resulting from Japan’s current policy of prolonged suspension of its governmental recommendation of the HPV vaccine
Source: Sci Rep. 2020 Sep 29;10:15945. doi: 10.1038/s41598-020-73106-z (PMC7524737; doi:10.1038/s41598-020-73106-z)
Supplement: Supplementary file 1 — Supplementary file1 [file 41598_2020_73106_MOESM1_ESM.pptx]

## Slide 1
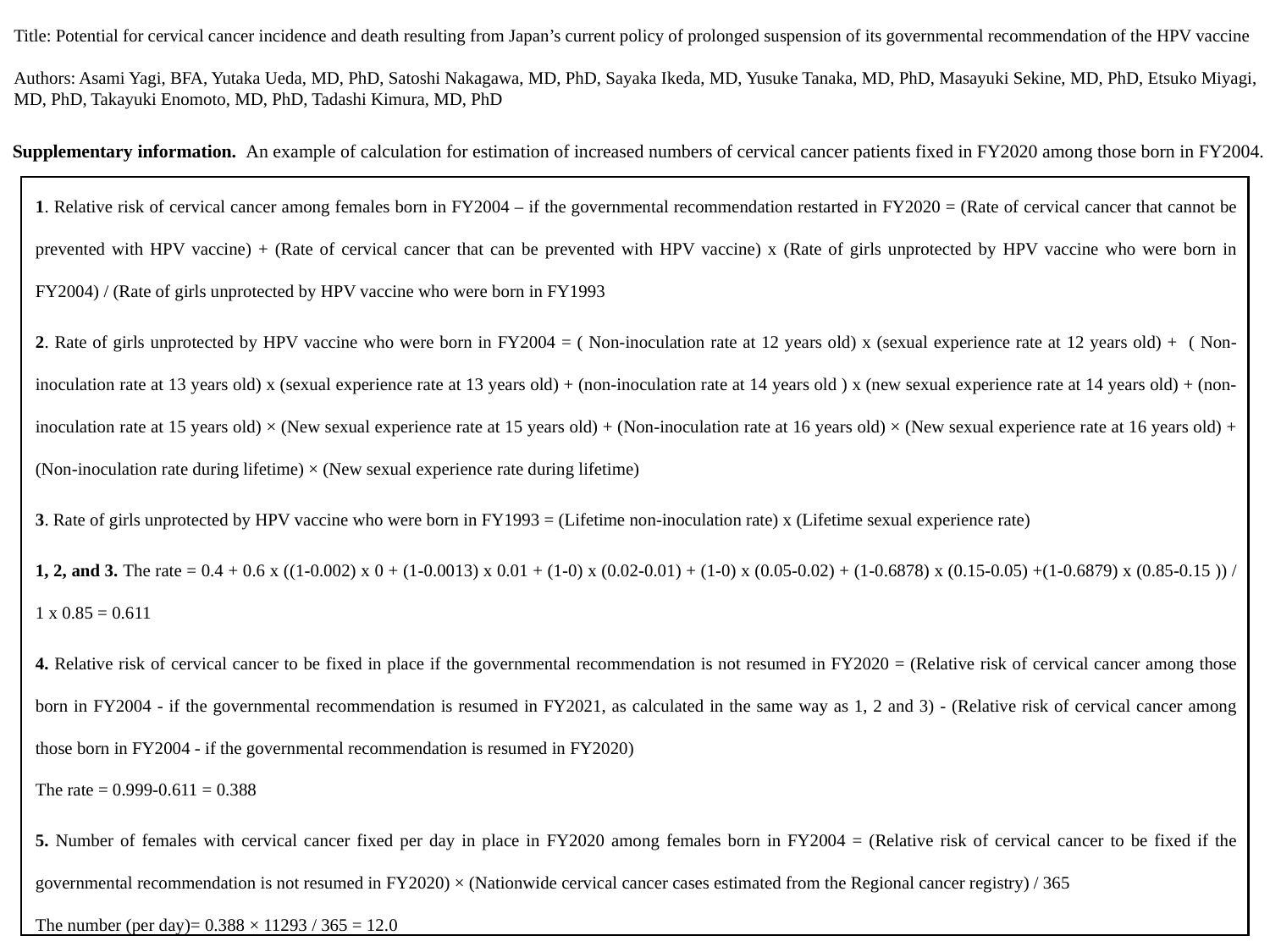

Title: Potential for cervical cancer incidence and death resulting from Japan’s current policy of prolonged suspension of its governmental recommendation of the HPV vaccine
Authors: Asami Yagi, BFA, Yutaka Ueda, MD, PhD, Satoshi Nakagawa, MD, PhD, Sayaka Ikeda, MD, Yusuke Tanaka, MD, PhD, Masayuki Sekine, MD, PhD, Etsuko Miyagi, MD, PhD, Takayuki Enomoto, MD, PhD, Tadashi Kimura, MD, PhD
Supplementary information. An example of calculation for estimation of increased numbers of cervical cancer patients fixed in FY2020 among those born in FY2004.
1. Relative risk of cervical cancer among females born in FY2004 – if the governmental recommendation restarted in FY2020 = (Rate of cervical cancer that cannot be prevented with HPV vaccine) + (Rate of cervical cancer that can be prevented with HPV vaccine) x (Rate of girls unprotected by HPV vaccine who were born in FY2004) / (Rate of girls unprotected by HPV vaccine who were born in FY1993
2. Rate of girls unprotected by HPV vaccine who were born in FY2004 = ( Non-inoculation rate at 12 years old) x (sexual experience rate at 12 years old) + ( Non-inoculation rate at 13 years old) x (sexual experience rate at 13 years old) + (non-inoculation rate at 14 years old ) x (new sexual experience rate at 14 years old) + (non-inoculation rate at 15 years old) × (New sexual experience rate at 15 years old) + (Non-inoculation rate at 16 years old) × (New sexual experience rate at 16 years old) + (Non-inoculation rate during lifetime) × (New sexual experience rate during lifetime)
3. Rate of girls unprotected by HPV vaccine who were born in FY1993 = (Lifetime non-inoculation rate) x (Lifetime sexual experience rate)
1, 2, and 3. The rate = 0.4 + 0.6 x ((1-0.002) x 0 + (1-0.0013) x 0.01 + (1-0) x (0.02-0.01) + (1-0) x (0.05-0.02) + (1-0.6878) x (0.15-0.05) +(1-0.6879) x (0.85-0.15 )) / 1 x 0.85 = 0.611
4. Relative risk of cervical cancer to be fixed in place if the governmental recommendation is not resumed in FY2020 = (Relative risk of cervical cancer among those born in FY2004 - if the governmental recommendation is resumed in FY2021, as calculated in the same way as 1, 2 and 3) - (Relative risk of cervical cancer among those born in FY2004 - if the governmental recommendation is resumed in FY2020)
The rate = 0.999-0.611 = 0.388
5. Number of females with cervical cancer fixed per day in place in FY2020 among females born in FY2004 = (Relative risk of cervical cancer to be fixed if the governmental recommendation is not resumed in FY2020) × (Nationwide cervical cancer cases estimated from the Regional cancer registry) / 365
The number (per day)= 0.388 × 11293 / 365 = 12.0
